# Supplementary material for: A Critical View of the Application of the APEX Software (Aqueous Photochemistry of Environmentally-Occurring Xenobiotics) to Predict Photoreaction Kinetics in Surface Freshwaters
Source: Molecules. 2019 Dec 18;25(1):9. doi: 10.3390/molecules25010009 (PMC7017383; doi:10.3390/molecules25010009)
Supplement: Supplementary file 1 [file molecules-25-00009-s001.zip › 3.2.4_gcc-4.4.0.zip!/3.2.4_gcc-4.4.0/3.2.4_gcc-4.4.0/doc/octave/html/interpreter/Bit-Manipulations.html]

Bit Manipulations - Untitled


Next: Logical Values,
Previous: Integer Data Types,
Up: Numeric Data Types


---

### 4.5 Bit Manipulations

Octave provides a number of functions for the manipulation of numeric
values on a bit by bit basis. The basic functions to set and obtain the
values of individual bits are `bitset` and `bitget`.

— Function File: x = **bitset** (a, n)  
— Function File: x = **bitset** (a, n, v)  
> Set or reset bit(s) n of unsigned integers in a.
> v = 0 resets and v = 1 sets the bits.
> The lowest significant bit is: n = 1
>
> ```
>           dec2bin (bitset (10, 1))
>           => 1011
> ```
>
>
>
>
> **See also:** bitand, bitor, bitxor, bitget, bitcmp, bitshift, bitmax.

— Function File: X = **bitget** (a,n)  
> Return the status of bit(s) n of unsigned integers in a
> the lowest significant bit is n = 1.
>
> ```
>           bitget (100, 8:-1:1)
>           => 0  1  1  0  0  1  0  0
> ```
>
>
>
>
> **See also:** bitand, bitor, bitxor, bitset, bitcmp, bitshift, bitmax.

The arguments to all of Octave's bitwise operations can be scalar or
arrays, except for `bitcmp`, whose k argument must a
scalar. In the case where more than one argument is an array, then all
arguments must have the same shape, and the bitwise operator is applied
to each of the elements of the argument individually. If at least one
argument is a scalar and one an array, then the scalar argument is
duplicated. Therefore

```
     bitget (100, 8:-1:1)
```

is the same as

```
     bitget (100 * ones (1, 8), 8:-1:1)
```

It should be noted that all values passed to the bit manipulation
functions of Octave are treated as integers. Therefore, even though the
example for `bitset` above passes the floating point value
`10`, it is treated as the bits `[1, 0, 1, 0]` rather than the
bits of the native floating point format representation of `10`.

As the maximum value that can be represented by a number is important
for bit manipulation, particularly when forming masks, Octave supplies
the function `bitmax`.

— Built-in Function: **bitmax** ()  
> Return the largest integer that can be represented as a floating point
> value. On IEEE-754 compatible systems, `bitmax` is `2^53 - 1`.

This is the double precision version of the functions `intmax`,
previously discussed.

Octave also includes the basic bitwise 'and', 'or' and 'exclusive or'
operators.

— Built-in Function: **bitand** (x, y)  
> Return the bitwise AND of non-negative integers.
> x, y must be in the range [0,bitmax]
>
>
>
> **See also:** bitor, bitxor, bitset, bitget, bitcmp, bitshift, bitmax.

— Built-in Function: **bitor** (x, y)  
> Return the bitwise OR of non-negative integers.
> x, y must be in the range [0,bitmax]
>
>
>
> **See also:** bitor, bitxor, bitset, bitget, bitcmp, bitshift, bitmax.

— Built-in Function: **bitxor** (x, y)  
> Return the bitwise XOR of non-negative integers.
> x, y must be in the range [0,bitmax]
>
>
>
> **See also:** bitand, bitor, bitset, bitget, bitcmp, bitshift, bitmax.

The bitwise 'not' operator is a unary operator that performs a logical
negation of each of the bits of the value. For this to make sense, the
mask against which the value is negated must be defined. Octave's
bitwise 'not' operator is `bitcmp`.

— Function File: **bitcmp** (a, k)  
> Return the k-bit complement of integers in a. If
> k is omitted `k = log2 (bitmax) + 1` is assumed.
>
> ```
>           bitcmp(7,4)
>           => 8
>           dec2bin(11)
>           => 1011
>           dec2bin(bitcmp(11, 6))
>           => 110100
> ```
>
>
>
>
> **See also:** bitand, bitor, bitxor, bitset, bitget, bitcmp, bitshift, bitmax.

Octave also includes the ability to left-shift and right-shift values bitwise.

— Built-in Function: **bitshift** (a, k)  
— Built-in Function: **bitshift** (a, k, n)  
> Return a k bit shift of n-digit unsigned
> integers in a. A positive k leads to a left shift.
> A negative value to a right shift. If n is omitted it defaults
> to log2(bitmax)+1.
> n must be in the range [1,log2(bitmax)+1] usually [1,33]
>
> ```
>           bitshift (eye (3), 1)
>           =>
>
> 2 0 0
>           0 2 0
>           0 0 2
>           
>           bitshift (10, [-2, -1, 0, 1, 2])
>           => 2   5  10  20  40
> ```
>
>
>
>
> **See also:** bitand, bitor, bitxor, bitset, bitget, bitcmp, bitmax.

Bits that are shifted out of either end of the value are lost. Octave
also uses arithmetic shifts, where the sign bit of the value is kept
during a right shift. For example

```
     bitshift (-10, -1)
     => -5
     bitshift (int8 (-1), -1)
     => -1
```

Note that `bitshift (int8 (-1), -1)` is `-1` since the bit
representation of `-1` in the `int8` data type is `[1, 1,
1, 1, 1, 1, 1, 1]`.
